# Hepato-bronchial fistula secondary to perforated sigmoid diverticulitis: a case report

**DOI:** 10.1186/s13256-017-1270-y

**Published:** 2017-04-13

**Authors:** Jun Sunny Yin, Shaylan Govind, Daniele Wiseman, Richard Inculet, Raymond Kao

**Affiliations:** 1 Schulich School of Medicine & Dentistry, University of Western Ontario, London, ON Canada; 2 Department of Medical Imaging, London Health Sciences Centre, University of Western Ontario, London, ON Canada; 3 Division of Thoracic Surgery, Department of Surgery, London Health Sciences Centre, University of Western Ontario, London, ON Canada; 4 Division of Critical Care, Department of Medicine, London Health Sciences Centre, Western University, London, ON Canada

**Keywords:** Diverticulitis, Hepatic abscess, Hepato-bronchial, fistula, Case report

## Abstract

**Background:**

Patients with diverticulitis are predisposed to hepatic abscesses via seeding through the portal circulation. Hepatic abscesses are well-documented sequelae of diverticulitis, however instances of progression to hepato-bronchial fistulization are rare. We present a case of diverticulitis associated with hepatic abscess leading to hepato-bronchial fistulization, which represents a novel disease course not yet reported in the literature.

**Case Presentation:**

A 61-year-old Caucasian man presented with a history of unintentional weight loss and dyspnea both at rest and with exertion. He had a significant tobacco and alcohol misuse history. A massive right-sided pleural effusion was found on chest X-ray, which responded partially to chest tube insertion. A computed tomography scan of his thorax confirmed the presence of innumerable lung abscesses as well as a complex pleural effusion. An indeterminate tiny air pocket at the dome of the liver was also noted. A follow-up computed tomography scan of his abdomen revealed a decompressed hepatic abscess extending into the right pleural space and the right lower lobe. A sigmoid-rectal fistula was also revealed with focal colonic thickening, presumed to be the sequelae of remote or chronic diverticulitis. An interventional radiologist inserted a percutaneous drain into the decompressed hepatic abscess and the instillation of contrast revealed immediate filling of the right pleural space, lung parenchyma, and bronchial tree, confirming a hepato-bronchial fistula. After two concurrent chest tube insertions failed to drain the remaining pleural effusion completely, surgical lung decortication was conducted. Markedly thickened pleura were seen and a significant amount of gelatinous inflammatory material was debrided from the lower thoracic cavity. He recovered well and was discharged 10 days post-thoracotomy on oral antibiotics. The percutaneous liver abscess tube was removed 3 weeks post-discharge from hospital after the drain check revealed that the fistula and abscess had entirely resolved.

**Conclusions:**

Refractory right-sided pleural effusion combined with constitutional symptoms should alert clinicians to search for possible hepatic abscess, especially in the context of diverticulitis. The rupture of an untreated hepatic abscess could lead to death from profound sepsis or rarely, as in this case, a hepato-bronchial fistula. Timely investigation and a multidisciplinary treatment approach can lead to improved patient outcomes.

## Background

Diverticulosis is a common condition where colonic wall weakness and intraluminal pressures predispose to formation of outpouching in the colonic wall, termed diverticula [1, 2]. It affects 5% of individuals under the age of 40 and 65% of those aged 65 and older [3]. Most are asymptomatic and lack leukocytosis, but may present with nonspecific complaints such as lower abdominal pain, bloating or constipation [1].

Diverticulitis, however, may arise when stool collects in and blocks the entrance of diverticula. This can lead to inflammation, mucous collection, and bacterial overgrowth causing distention and ischemia [4]. The treatment for diverticulitis includes bowel rest and antibiotics, which is sufficient to resolve symptoms in 75% of cases [5]. However, complications may include microperforations with pericolic abscess formation as well as macroperforations with gut-to-gut fistulization and abscess formation throughout the abdomen [1, 6, 7]. Abscesses may be treated with antibiotics and, depending on size, drainage may be indicated. Fistula generally requires surgical intervention [5]. Our case demonstrates an atypical initial presentation of diverticulitis in addition to a novel disease course, where diverticulitis lead to hepatic abscess formation, which then ruptured causing hepato-bronchial fistulization.

## Case presentation

A 61-year-old Caucasian man presented to our emergency department with 20 pounds of unintentional weight loss over 2 months followed by a 2-week history of worsening shortness of breath, epigastric tenderness, and bilateral pitting leg edema. He had no symptoms of fever, cough, phlegm production, diaphoresis or night sweats. He had a 40 pack-year smoking history and 40-year history of alcohol misuse, but no significant past medical history or family history of significant medical illness. Later in the course of his current presentation, he admitted to 6 months’ history of intermittent abdominal pain and diarrhea. He had attributed this to his use of alcohol and he did not seek medical care.

On physical examination, he was emaciated and afebrile with a heart rate of 118 beats per minute and a blood pressure of 103/70 mmHg. His jugular venous pressure was elevated at 5 cm above the sternal angle. His abdomen was not distended but tender to light palpation in the epigastrium. He was not jaundiced. Leg pitting edema was noted from his feet up to the distal thirds of his tibia bilaterally. Auscultation of the lung fields revealed significantly decreased air entry at the base of the right lung. His oxygen saturation on room air was found to be 85%. With supplemental oxygen delivered by nasal prongs at 2L/minute he maintained an oxygen saturation of 94%.

The admission blood work is presented in Table 1. Results showed an elevated leukocyte count of 24.0 × 10^9^/L, predominately neutrophils, elevated thrombocyte count of 894 × 10^9^/L and low hemoglobin 79 g/L with a hematocrit of 27%. Liver enzyme test results revealed elevated alkaline phosphatase 197 U/L, elevated gamma glutamyl transferase (ϒGt) 121 U/L, international normalized ratio (INR) elevated at 1.4 and C-reactive protein (CRP) elevated at 262.5 mg/L. There was a significant drop in his albumin level, at 24 g/L. Serology results did not demonstrate any evidence of hepatitis B infection. Additionally, no evidence of hepatitis B immunity was found. Hepatitis C antibodies were non-reactive.

**Table 1:** Admission hematology and biochemistry data

	Measured value	Reference range
Hematology		
Leukocyte count	24.0 × 10^9^/L	4.0 × 10^9^/L – 10.0 × 10^9^/L
Hemoglobin	79 g/L	135 g/L – 170 g/L
Thrombocytes	894 × 10^9^/L	150 × 10^9^/L – 400 × 10^9^/L
Hematocrit	27%	40% – 51%
Mean corpuscular volume (MCV)	80.8 fL	79fL – 97fL
Enzymes		
Alanine aminotransferase (ALT)	16 U/L	<40 U/L
Aspartate aminotransferase (AST)	9 U/L	<39 U/L
Alkaline phosphatase	197 U/L	40 U/L – 129 U/L
Gamma glutamyl transferase (ϒGt)	121 U/L	<60 U/L
Lactate dehydrogenase (LDH)	90 U/L	<224 U/L
Coagulation		
International normalized ratio (INR)	1.4	0.9sec – 1.1sec
Partial thromboplastin time (PTT)	29	23sec – 32sec
Immunology		
C-reactive protein (CRP)	262.6 mg/L	<5.0mg/L
Serum electrolytes		
Sodium	133 mmol/L	135 mmol/L – 145 mmol/L
Potassium	4.5 mmol/L	3.5 mmol/L – 5.0 mmol/L
Chloride	96 mmol/L	98 mmol/L – 107 mmol/L
Bicarbonate	26 mmol/L	22 mmol/L – 29 mmol/L
Urea	3.7 mmol/L	<8.2 mmol/L
Creatinine	56 μmol/L	62 μmol/L – 120 μmol/L
Albumin	24 g/L	35 g/L – 52 g/L
Calcium	2.21 mmol/L	2.15 mmol/L – 2.55 mmol/L
Magnesium	0.8 mmol/L	0.65 mmol/L – 1.05 mmol/L
Phosphate	1.58 mmol/L	0.80 mmol/L – 1.33 mmol/L
Total bilirubin	11.9 μmol/L	3.4 μmol/L – 17.1 μmol/L
Random glucose	7.1 mmol/L	3.4 mmol/L – 11.0 mmol/L

**Fig. 1:**
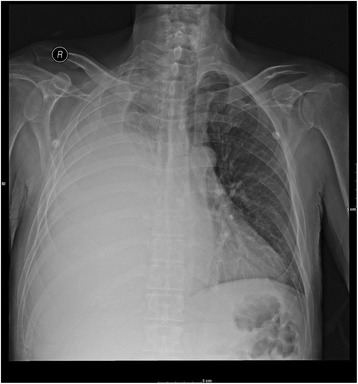
Chest X-ray shows almost complete opacification with a large right pleural effusion and consolidated right lower lobe

The admission chest X-ray, Fig. 1, revealed a large right lung pleural effusion with near complete opacification associated with mass effect and mild mediastinal shift toward the left hemithorax. A thoracentesis removed 1.5 L of yellow cloudy fluid from the right hemithorax in the emergency room. The pleural fluid analysis revealed an exudative effusion with a fluid lactate dehydrogenase of 256 U/L, nucleated cell count of 14.3 × 10^9^ with 86% being neutrophils, and fluid to serum amylase ratio of 1:2. Light’s criteria were assessed and consistent for exudative effusion, Table 2. The serum effusion albumin gradient (SEAG) was measured at 5g/L (serum albumin 24g/L – effusion albumin 19g/L), which is consistent with exudative effusion. The fluid was also sent for Gram stain and microbiological culture.

**Table 2:** Light’s criteria for exudative effusion

Light’s criteria	Value	Exudative range
Pleural fluid protein to serum protein ratio	0.81	>0.5
Pleural fluid lactate dehydrogenase (LDH) to serum LDH ratio	2.8	>0.6
Pleural fluid LDH level	256 U/L	>149 U/L

Our patient was admitted to the internal medicine ward and piperacillin-tazobactam 3.375 g intravenously every 6 hours was initiated. A right-sided Wayne catheter chest tube was inserted with ultrasound guidance the following day; however chest X-rays post chest tube insertion did not show any significant decrease in effusion size. An esophagogastroduodenoscopy (EGD) and colonoscopy were performed to help determine the cause of the patient’s anemia. The EGD did not identify a source of bleeding, but the colonoscopy revealed an impassable diverticular stricture encountered at 25 cm from the sigmoid colon.

**Fig. 2:**
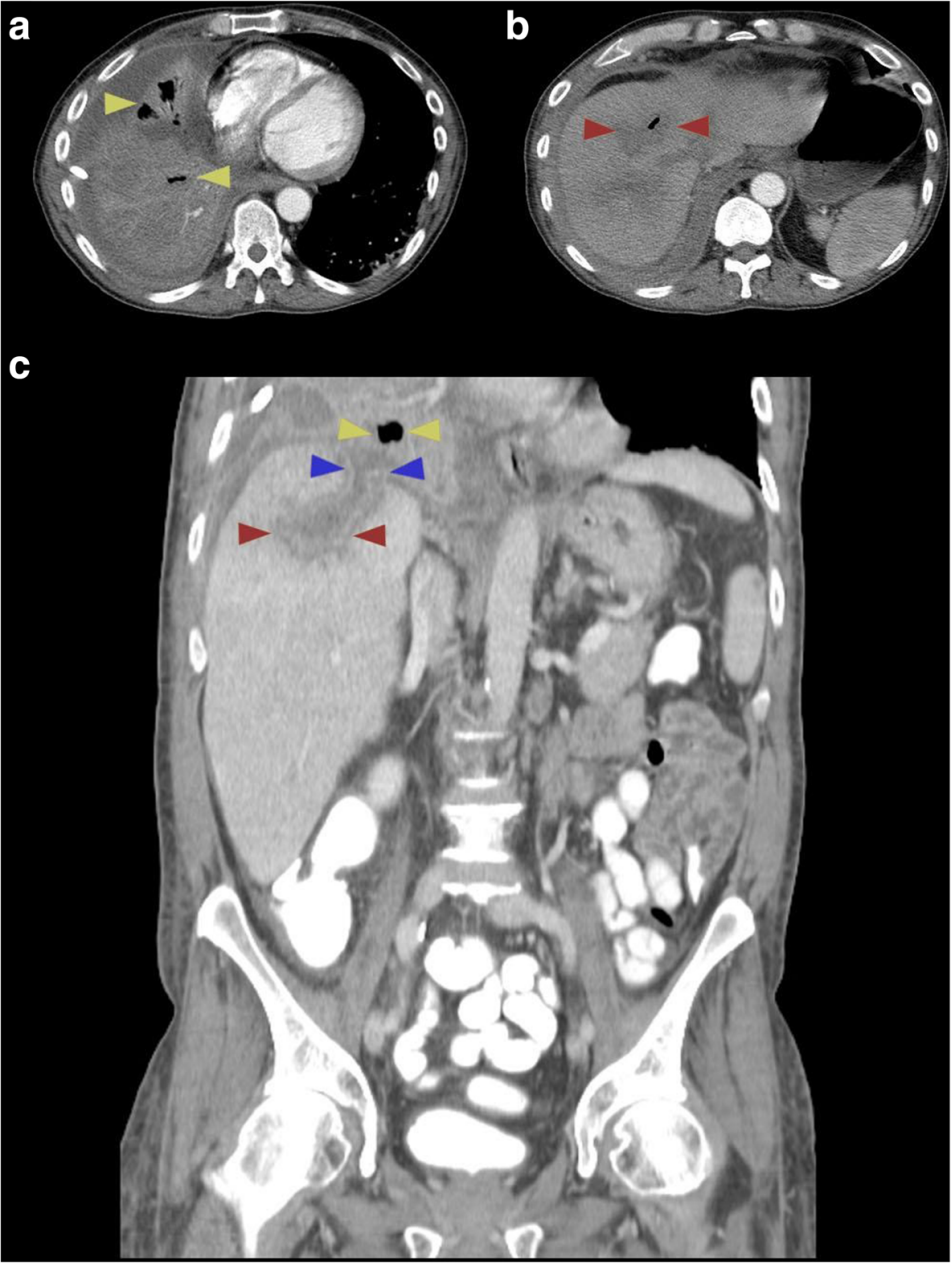
**a** Computed tomography thorax scan with contrast reveals multiple air-filled lung abscesses in the right lung lobe (*yellow arrows*). **b** The air-filled lung abscess is contiguous with a small, collapsed area of hypoattenuation within liver parenchyma with central air pocket in segment 8 of the liver (*red arrows*). **c** Computed tomography abdomen and pelvis scan with portal venous phase intravenous contrast clarified a continuous track (*blue arrows*) from a collapsed, peripherally enhancing liver abscess (*red arrows*) extending across the diaphragm and in continuity with right lower lobe lung abscesses (*yellow arrows*)

**Fig. 3:**
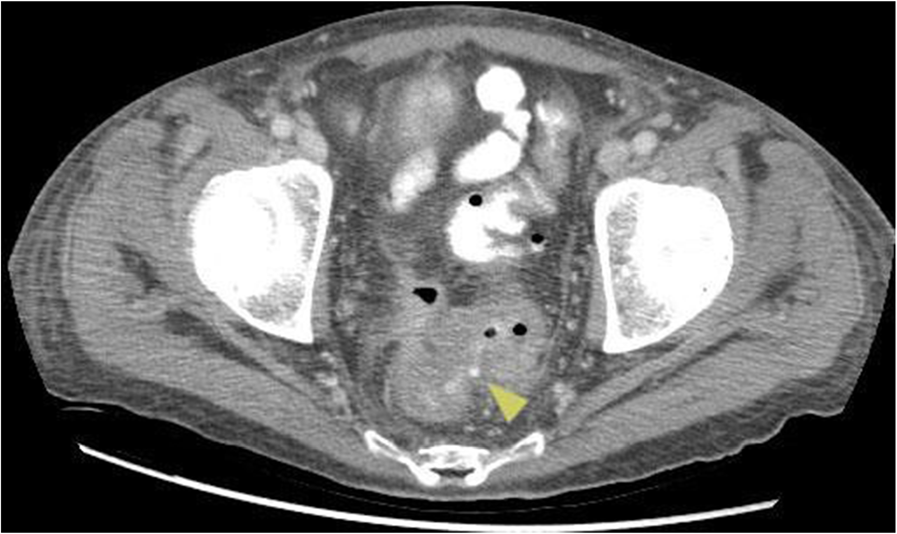
Computed tomography pelvis scan shows focal rectosigmoid thickening with small fistula from sigmoid to rectum (*yellow arrow*) in the region of inflamed diverticula suggesting complicated diverticulitis. The fistula formation suggests an element of chronicity

A computed tomography (CT) scan of our patient’s thorax demonstrated a tiny gas and fluid collection in the right lobe of the liver, suggestive of decompressed hepatic abscess with extension through the right pleural space and into the right lower lobe of the lung, Fig. 2. A CT scan of his abdomen confirmed a ruptured intrahepatic abscess extending across the diaphragm into the right pleura and into small right lower lobe abscesses. Furthermore, a complicated sigmoid colon diverticulitis with a fistula to the rectum was also seen, Fig. 3.

**Fig. 4:**
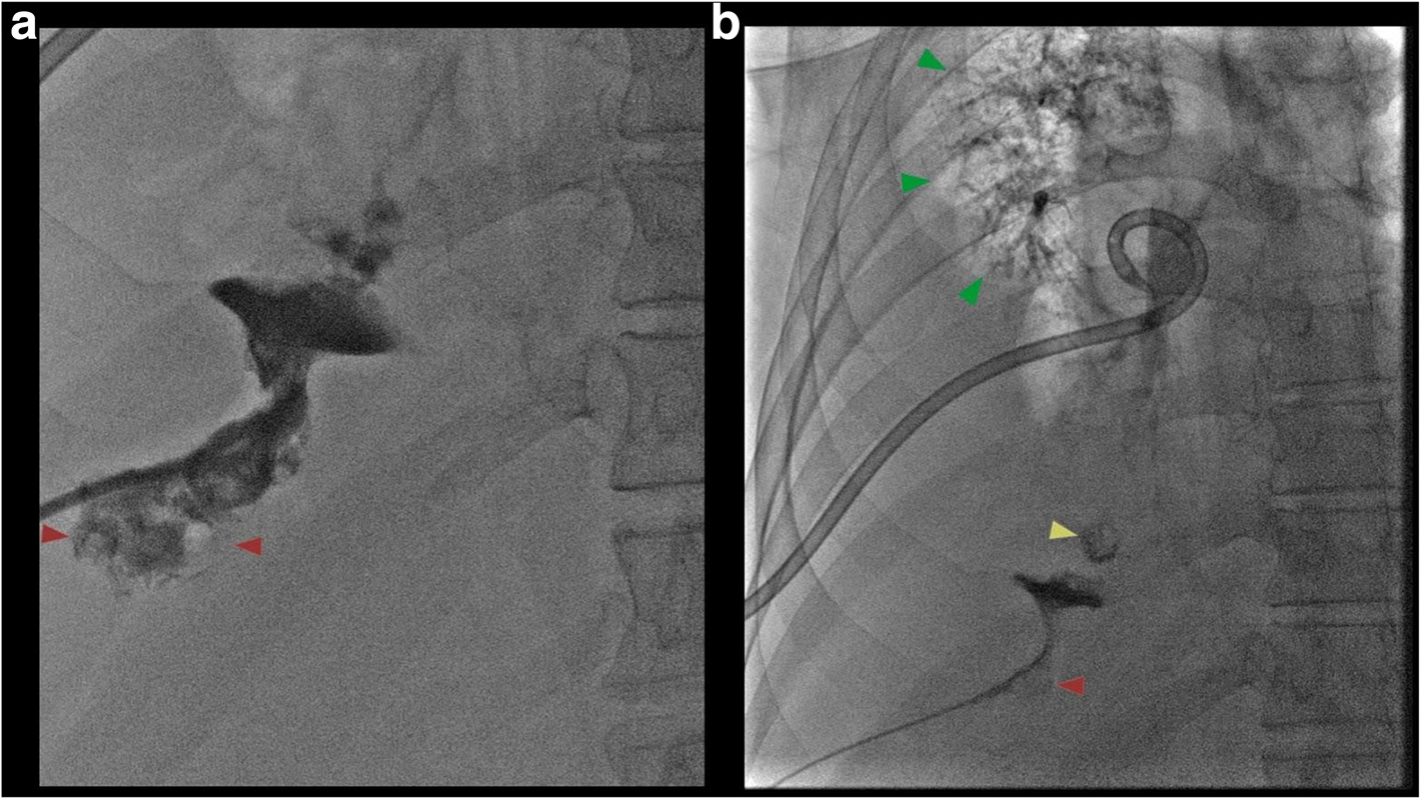
**a** Fluoroscopic view shows contrast in collapsed liver abscess (*red arrows*) extending upward through the capsule. **b** Further fluoroscopic view shows contrast tracking from the liver abscess in segment 8 (*red arrow*), through the right hemidiaphragm into a contained collection in the pleura (*red arrow*) and then into small lung abscess (*yellow arrow*) then filling the right bronchial tree (*green arrows*)

At this time, the general and thoracic surgery services were consulted. A decision was made to request the interventional radiology service to place a percutaneous drain to clear the remaining intrahepatic abscess. During the procedure, fluoroscopic contrast was instilled into the abscess drain and was found to track through the right hemidiaphragm into the right lung pleural space and bronchi, Fig. 4. This resulted in a sudden drop in the patient’s oxygen saturation, which resolved when the contrast was evacuated from the bronchi. A new chest tube was inserted to drain the remaining pleural fluids. Despite the intrahepatic drain and two chest tubes in situ, the patient’s pleural effusions failed to drain completely. The thoracic surgery team subsequently decided that lung decortication and exploratory bronchoscopy was indicated for the patient. The microbiological culture of the pleural fluid grew penicillin-sensitive *Streptococcus anginosus*, but our patient’s antibiotic regimen was not changed in order to maintain coverage for possible anaerobic and Gram-negative enteric microbes.

Our patient was taken to the operating room, where bronchoscopy revealed clear airways with no perforations. A thoracotomy was then performed to expose the pleura, which was found to be markedly thickened. The pleura were incised and a large amount of gelatinous material was removed from the pleural cavity. Extensive decortication of the right lung resulted in significantly improved intraoperative lung inflation. A biopsy of the lung abscess was also taken. Four chest tubes were placed intraoperatively to ensure effective drainage of the pleural cavity. Our patient tolerated the procedure well and substantial lung re-expansion was observed on chest X-rays in the days following the procedure. A subsequent drain check performed by an interventional radiologist, showed a residual cavity in the right lobe of the liver and that the fistula to the pleural space was still patent. However, no continuity to the lung abscess or bronchi was observed. General surgery also opted not to surgically remove the fistula as it was felt that it would close on its own given the effective drainage that had been achieved.

Ten days following his operation, our patient was discharged home with his hepatic abscess drain still *in situ* and with a 21-day course of ciprofloxacin 500 mg and metronidazole 500 mg, both orally and twice daily. An abscess check performed 3 weeks later found that the abscess had resolved without complications and the percutaneous drain was removed.

The general surgery service re-assessed our patient approximately 1 month post-discharge from hospital. Our patient reported he was feeling well and denied experiencing any abdominal pain or other concerning gastrointestinal symptoms. Because of this complex diverticulitis with an increased the risk of future bacterial seeding, a lower anterior resection of the affected colon and rectum was offered to the patient. Our patient agreed to have this procedure performed laparoscopically, however significant adhesions were found intraoperatively in the pelvis preventing easy mobilization of the sigmoid colon. Subsequently, the operation was converted to an open laparatomy. The segment of colon containing the fistula was identified and resected. A tension-free end-to-end anastomosis was then created without difficulty. Our patient tolerated the procedure well and was discharged from hospital 5 days following the operation. At follow-up appointments at 1 week and 1 month following his discharge, our patient again reported that he was feeling well and denied experiencing any abdominal pain or other gastrointestinal symptoms.

## Discussion

We report the case of a 61-year-old man with perforated diverticulitis resulting in sigmoid-rectal fistulization, and hepatic abscess formation with subsequent hepatic-bronchial fistulization. To the best of our knowledge, this is the first reported case and description of a hepatic-bronchial fistulization secondary to diverticulitis.

Abdominal complications of diverticulitis including perforation, bleeding, obstruction, and fistula formation are often due to bacterial overgrowth [4]. The organisms commonly implicated include *Escherichia coli*, *Streptococcus* spp., *Bacteroides* spp., *Peptostreptococcus*, *Clostridium*, and *Fusobacterium* spp. [8]. Bowel microperforations can cause pericolic abscess formation, while macroperforations can cause gut-to-gut fistulization and abscess formation throughout the abdomen, including hepatic abscesses [1, 6, 7].

The most common route of bacterial seeding to the liver is hematogenous spread via the portal circulation. The right lobe in particular is involved in 72% of hepatic abscesses [6] as it receives the majority of portal circulation [9, 10]. The intestinal microorganisms are able to cross the gut-vascular barrier and enter the venous circulation either via various virulence factors [11], or via intestinal perforations [12] similar to those caused by diverticulitis. The formation of hepatic abscesses caused by intestinal perforations from diverticulitis has also been documented in the past [6, 7].

In our case, the sigmoid colon diverticulosis with focal thickening and a fistula to the rectum was discovered on abdominal CT scan. Subsequent investigations led to the discovery of a hepato-bronchial fistulization. The complication of hepatic-bronchial fistulae has been described as a rare complication of hepatic amebiasis [13], radiofrequency ablation for hepatocellular carcinoma [14], or as a surgical complication after cholecystectomy [15]. However, to the best of our knowledge hepatic-bronchial fistulization from ruptured hepatic abscess resultant from a chronic perforated sigmoid colon diverticulosis has not yet been reported. As such, this case demonstrates both an atypical presentation of diverticulitis, as well as a novel course of this disease.

We hypothesize that our patient suffered a perforation in the sigmoid colon due to diverticulitis, as evidenced by the sigmoid-rectal fistula, Fig. 3. This mechanical disruption allowed intestinal microorganisms to translocate into the portal circulation, seeding the liver. The subsequent hepatic abscess formation in the right hepatic lobe, Fig. 2, produced inflammation that eventually ruptured and eroded through the diaphragm and penetrated into the right pleural cavity and right lobe of the lung. As a result, a massive pleural effusion and empyema that triggered our patient’s respiratory distress and prompted his presentation to hospital, Fig. 1. The severe pleural inflammation and infection, as evidenced by the markedly thickened pleura and large volume of loculated infected material, caused fistulization to the bronchi of the right lower lobe. This final sequela was clearly demonstrated by the spread of contrast material into the bronchial tree when it was instilled into the hepatic abscess, Fig. 4. The pericolic abscess formation, hepatic abscess formation, and hepatic-bronchial fistulization have all been described occurring as independent clinical phenomena; however, our case clearly demonstrates a novel stepwise clinical course initially triggered by probable remote, untreated perforated diverticulitis.

The initial presentation in our emergency department with epigastric tenderness could have been referred pain from a right upper quadrant liver pathology and/or pathology in the right lower lobe of the lung. We believe during the stepwise care our patient received, the chest X-ray revealed a large pleural effusion which focused the provider on the lung pathology and the epigastric pain associated with anemia focused the provider on the possibility of an upper gastrointestinal bleed. This case clearly illustrates the utility of Ishikawa diagrams in delineating possible etiology when presented with gastroenterological and pulmonary symptoms. In our case this was expanded and refined based on other associated symptoms followed by radiological and laboratory examinations [16].

The lack of fever, negative blood cultures, and negative hepatic abscess fluid cultures were certainly unexpected. These negative findings could be due to concurrent antibiotic therapy or imperfect culture sensitivity, both of which are known factors associated with culture-negative infections [17]. The pleural fluid drained did grow *Streptococcus anginosus*, a member of the Streptococcus milleri group, known for their tendency to cause abscesses [18]. In particular, *S. anginosus* is isolated significantly more frequently than other group members in the gastrointestinal and genitourinary systems [19], adding further support for an intestinal source of infection in this case.

Pyogenic hepatic abscesses are treated with aspiration of the abscess including aspirate cultures to determine the causative organisms and antibiotic susceptibilities. Concurrent empiric intravenous antibiotics covering enterobacteriaceae and anaerobes are also utilized [20]. The antibiotic therapy can be narrowed once susceptibilities are available, and should continue for 4–6 weeks [20]. In cases of hepatic-bronchial fistulization, management of the hepatic abscess is the same as described above, and this generally also resolves the respiratory symptoms without additional therapy [12, 13, 15]. In cases with refractory pleural effusions or empyema, the management begins with chest tube drainage of the complicated pleural fluids [21]. Surgical decortication can be employed for non-refractory cases [21]. In our case, the appropriate empiric intravenous antibiotics were started and the hepatic abscess was drained. To address his stage III [22] empyema, thoracentesis was performed and two chest tubes were placed. Our patient demonstrated little improvement following these interventions, and lung decortication was eventually required.

Our patient’s behavior with respect to seeking medical care is another important aspect of this case. Our patient described 20 pounds of unintentional weight loss over 2 months as well as a 6-month history of intermittent abdominal pain and diarrhea. However, despite these very concerning symptoms, our patient did not seek any medical attention. It was not until his shortness of breath began to interfere with his daily activities that he decided to seek medical attention. This case illustrates the importance of assessing patients’ healthcare-seeking behavior. When patients are found to seek healthcare very infrequently, it should raise a clinician’s index of suspicion for an acute exacerbation of an undiagnosed chronic condition.

Consultations to both the radiology service for the CT thorax scan and the gastroenterology service for endoscopy to rule out a gastrointestinal source of bleeding were done at the same time. The gastroenterology service was able to review our patient first, prior to the CT thorax scan being obtained. Subsequently, the CT thorax scan revealed a tiny gas and fluid collection in the right lobe of the liver, suggestive of a decompressed hepatic abscess with extension through the right pleural space and into the right lower lobe of the lung. On review of the management our patient received, it is possible that because his anemia and respiratory findings were so significant the care providers were distracted from investigating the 6-month history of intermittent abdominal pain and diarrhea that he endorsed at his initial presentation. These symptoms are indeed suggestive of an abdominal source such as diverticulitis and as such an abdominal CT scan could have been performed earlier. This would have led to faster discovery of the etiology of his underlying pathology.

Additionally, a carcinoembryonic antigen (CEA) should have been measured after the colonic stricture was discovered as an elevated CEA suggesting malignancy would have significantly changed his management. Fortunately, the pathology report on the colonic specimen from his lower anterior resection did not note any signs of malignancy.

## Conclusions

Hepatic abscess leading to hepato-bronchial fistula is a rare cause of massive pleural effusion and empyema that can lead to poor outcomes if the diagnosis is missed or delayed. Hepato-bronchial fistulization from a hepatic abscess should be considered when patients present with refractory pleural effusion, constitutional symptoms, and intestinal disease such as diverticulitis. Timely CT imaging of the thorax and abdomen is key in making the diagnosis, and for best patient outcome, a multidisciplinary approach involving disciplines such as pulmonary medicine, interventional radiology, and thoracic surgery is warranted.

## Abbreviations

CT: Computed tomography
EGD: Esophagogastroduodenoscopy
